# Heterogeneity in immune cell composition is associated with *Mycobacterium tuberculosis* replication at the granuloma level

**DOI:** 10.3389/fimmu.2024.1427472

**Published:** 2024-08-26

**Authors:** Sarah K. Cooper, David Forrest Ackart, Faye Lanni, Marcela Henao-Tamayo, G. Brooke Anderson, Brendan K. Podell

**Affiliations:** ^1^ Mycobacteria Research Laboratories, Department of Microbiology, Immunology, and Pathology, Colorado State University, Fort Collins, CO, United States; ^2^ Phoenix Immune Mechanisms of Protection Against Tuberculosis Center, Seattle, WA, United States; ^3^ Department of Environmental and Radiological Health Sciences, Colorado State University, Fort Collins, CO, United States; ^4^ Consortium for Applied Microbial Metrics, Aurora, CO, United States

**Keywords:** *Mycobacterium*, tuberculosis, granuloma, immunohistochemistry (IHC), *in situ* hybridization (ISH), image analysis

## Abstract

The control of bacterial growth is key to the prevention and treatment of tuberculosis (TB). Granulomas represent independent foci of the host immune response that present heterogeneous capacity for control of bacterial growth. At the whole tissue level, B cells and CD4 or CD8 T cells have an established role in immune protection against TB. Immune cells interact within each granuloma response, but the impact of granuloma immune composition on bacterial replication remains unknown. Here we investigate the associations between immune cell composition, including B cell, CD4, and CD8 T cells, and the state of replicating *Mycobacterium tuberculosis* (*Mtb)* within the granuloma. A measure of ribosomal RNA synthesis, the RS ratio®, represents a proxy measure of *Mtb* replication at the whole tissue level. We adapted the RS ratio through use of *in situ* hybridization, to identify replicating and non-replicating *Mtb* within each designated granuloma. We applied a regression model to characterize the associations between immune cell populations and the state of *Mtb* replication within each respective granuloma. In the evaluation of nearly 200 granulomas, we identified heterogeneity in both immune cell composition and proportion of replicating bacteria. We found clear evidence of directional associations between immune cell composition and replicating *Mtb*. Controlling for vaccination status and endpoint post-infection, granulomas with lower CD4 or higher CD8 cell counts are associated with a higher percent of replicating *Mtb*. Conversely, changes in B cell proportions were associated with little change in *Mtb* replication. This study establishes heterogeneity across granulomas, demonstrating that certain immune cell types are differentially associated with control of *Mtb* replication. These data suggest that evaluation at the granuloma level may be imperative to identifying correlates of immune protection.

## 1 Introduction

Tuberculosis (TB) granulomas are isolated and multifocal structures formed by the host immune response to combat *Mycobacterium tuberculosis* (*Mtb*) infection. Their role is to contain and clear the bacteria, thereby preventing further bacterial replication, disease, and transmission. However, granuloma formation plays contradictory roles in TB disease; while the formation of granulomas control *Mtb* replication, they also allow *Mtb* to persist and replicate (1). There is considerable heterogeneity in the pathology, immune cell composition and structure of granulomas (2, 3). The underlying immune mechanisms that contribute to control of *Mtb* infection across the heterogeneous population of granulomas are poorly understood. Determining natural and vaccine-mediated immune protection at the granuloma level can provide key insights into developing more effective vaccines and therapeutics.

Heterogeneity has been described in granulomas across multiple species including human, non-human primate (NHP), rabbit, guinea pig, and some strains of mice. Granulomas within the same host may appear simultaneously active and inactive by PET-CT imaging in both humans and non-human primate (NHP) models (4–6). Autopsies of human subjects show a wide diversity in granuloma morphology across disease states (7, 8), which is also reflected by NHP models (9). Some NHP granulomas demonstrate evidence of resolution by histopathology and PET-CT imaging, while others in the same host, remain active (10). While substantial heterogeneity exists across many of commonly used mouse strains (11–13), the guinea pig as a small rodent model uniquely demonstrates central caseous necrosis and granuloma organization that is characteristic of human TB (14–16). When evaluated in individual granulomas, the culturable bacterial burden used to assess protection has revealed a wide range of heterogeneity consistent with these pathology observations (17). Collectively, evidence across multiple model species and human subjects supports the presence of substantial heterogeneity in granuloma composition and function.

CD4 T cells represent the cornerstone of antimycobacterial immunity. Murine studies eliminating CD4 T cells from the immune response have demonstrated loss of protection, based on changes in culturable burden from infected lung tissue (18, 19). This protection includes both IFNγ dependent and independent mechanisms derived from CD4 T cells (18). These CD4 T cells are present in multiple states of differentiation, polarization, effector functions or memory phenotypes (20, 21). Recently, a role for B cell derived antibody and CD8 T cells has been shown in correlates of immune protection (22). Antigen-specific antibodies may exert multiple effector functions in response to *Mtb* infection including antibody-dependent cellular cytotoxicity, opsonization and facilitated phagocytosis, as well as fixation and activation of complement (23, 24). Although functions of CD8 T cells are less understood, these cells are critical to control of TB progression with links to granzymes and cytotoxic function (25–27). Although important to immune protection, the contribution of these adaptive immune cell types to granuloma success or failure remains poorly understood, primarily due to limited methods that simultaneously measure both host cell populations and individual bacterial response within a single granuloma.

Protection is typically determined by measuring differences in culturable *Mtb* from tissue via colony forming units (CFU). Although this offers important information on bacterial burden, it only measures *Mtb* capable of growth on solid agar and as such cannot provide a specific location from where the bacteria were derived. Location is particularly important in considering granuloma heterogeneity because granulomas within a single animal may exist in differing states or have differing degrees of capacity for bacterial control. Previously, observation of *Mtb* in granulomas of pathology specimens has been limited to use of either reporter strains, or cell-wall based staining through IHC or variations on the traditional acid-fast stain. These methods have limitations in both stability and reproducibility, as well as capacity to detect the total *Mtb* population (28–30). These measures also fail to provide information on the physiologic status or replicative response of the *Mtb* bacilli.

A novel adjunctive measure to CFU, the RS ratio®, measures the ratio of *Mtb* immature pre-rRNA relative to mature 23S rRNA, representing ongoing rRNA synthesis (31). We have previously shown that the RS ratio effectively serves as a proxy measure of bacterial replication status. By applying *in situ* hybridization (ISH) probes targeting these regions of rRNA, we have established the RS ratio ISH approach for detection of *Mtb* bacilli and their state of replication, thereby enabling investigation of both the host and pathogen response within a single granuloma. This approach can measure changes in response over time, and in response to vaccination, across states of both host cell populations and *Mtb* replication. Here, we measure CD4 T cell, CD8 T cell, and B cell populations using multiplexed fluorescent IHC simultaneously with the RS ratio ISH across granulomas of unvaccinated and BCG-vaccinated mice, demonstrating an association between granuloma immune cell composition and *Mtb* replication rates.

The heterogeneous nature of immune cell composition across granulomas, as demonstrated by this study, provides strong evidence that certain immune cells have a greater influence on the control of *Mtb* replication. These results emphasize the need for granuloma-focused investigations to advance the understanding of protective immunity against *Mtb* infection and inform novel vaccine and therapeutic strategies.

## 2 Materials and methods

### 2.1 Animals

Male and female C57BL/6 mice, 4–6 weeks of age, were purchased from The Jackson Laboratory. Mice were housed in a biosafety level 3 animal facility at Colorado State University (CSU), and all experimental protocols and procedures were performed in accordance with the CSU Institutional Animal Care and Use Committee under protocol number 1278. Mice were euthanized by CO2 inhalation at 56 or 114 days after exposure to *Mtb*.

### 2.2 Vaccination

Mice were mock-vaccinated with 50 μl of phosphate buffered saline (PBS) or vaccinated with the human clinical-grade Danish-1331 BCG vaccine, Lot #120005-C (AJ Vaccines), by intradermal injection at the tail base. Assuming a stock bacterial concentration of 2 x 10^6^ BCG organism per ml, as provided by the manufacturer, a targeted dose of 1 x 10^4^ CFU was administered in a 50 μl injection volume to each mouse.

### 2.3 *Mycobacterium tuberculosis* infection

150 days post-vaccination with BCG, mice were infected by low-dose aerosol with *Mtb* Erdman (BEI Resources) using a Glas-Col aerosol chamber targeting an exposure dose of 50–100 CFU, as previously described (32). Immediately post-exposure, three mice were euthanized and whole lung homogenate was plated on 7H11 agar plates to confirm aerosol delivery of expected exposure dose, achieving a mean exposure dose of 54 CFU per mouse.

### 2.4 Bacterial enumeration by CFU

At necropsy, the right caudal lung lobe of each mouse was collected and homogenized in PBS using a Bullet Blender Blue (Next Advance) device at speed 8 setting for 4 minutes, as previously described (33). Tissue homogenate was plated at serial 1:5 dilutions on 7H11 agar plates and final CFU counts were enumerated following 8 weeks of incubation.

### 2.5 Histopathology and image analysis

One third of the left lung lobe was collected at necropsy and immediately fixed in 4% paraformaldehyde for 48 hours before being paraffin embedded. 5μm tissue sections were cut and mounted on charged microscope slides, stained with hematoxylin and eosin (H&E), and scanned at 20X magnification using an Olympus VS120 scanning microscope, Hamamatsu ORCA-R2 camera, and Olympus VS-ASW 2.9 software available through the Experimental Pathology Facility at Colorado State University.

Visiopharm software was used for image analysis. Entire lung section regions of interest (ROI) were identified at 5X magnification with a custom algorithm that utilizes decision forest training to differentiate tissue from microscope slide based on color and area. An additional custom-made algorithm based on decision forest training and classification based on staining intensity, area, and morphological features at 20X magnification was created to automate granuloma detection. A final algorithm was used for quantification of area of both granulomas and healthy tissue within tissue section ROIs to calculate percent granuloma lesion burden. Identified granulomas and calculations received a final review by a pathologist (Podell BK) (34).

### 2.6 Multiplex fluorescent immunohistochemistry

Slides were deparaffinized and stained using a Leica Bond RXm automated slide stainer and associated Leica Bond reagents (**Supplementary Table 1**). Heat induced epitope retrieval (HIER) was performed on tissues with ER1 solution at 97°C for 30 minutes prior to quenching endogenous peroxidase activity with 3% hydrogen peroxide. Tissues were blocked using 2.5% Normal Goat Serum Blocking Solution (Vector Laboratories) then incubated with the following rat monoclonal primary antibodies: CD4 (1:100, clone 4SM95, catalog number 14–9766-82, eBioscience), CD8a (1:100, clone 4SM15, catalog number 14–0808-82, eBioscience), and CD45R/B220 (1:100, clone RA3–6B2, catalog number 103201 BD Biosciences). Sections were incubated with undiluted ImmPRESS mouse adsorbed goat anti-rat IgG-HRP polymer (Vector Laboratories) for primary antibody detection. Visualization of cell surface markers was accomplished using Opal 520 (1:200), Opal 570 (1:200), and TSA plus Cyanine 5 (1:1000) in 1X TSA Plus Automation Amplification Diluent (Akoya Biosciences). Antibody stripping was performed using HIER at 95°C and ER1 solution for 20 minutes (Leica Biosystems) between each antibody application in the following order: CD4 detected with Opal 520, CD8 detected with Opal 570, and B220 detected with Cyanine 5. Finally, nuclei were stained with Spectral DAPI (Akoya Biosciences). Whole stained lung sections were scanned at 20X magnification using an Olympus VS120 microscope, Hamamatsu ORCA-R2 camera, and Olympus VS-ASW 2.9 software at the Experimental Pathology Facility at Colorado State University. Exposure times were determined using the autoexposure function and adjusted to distinguish antibody detection of cell surface markers from erythrocyte background.

### 2.7 Multiplex fluorescent *in situ* hybridization

Slides cut in serial to those used for multiplex IHC were deparaffinized and targets hybridized using the RNAscope LS multiplex fluorescent reagents (Advanced Cell Diagnostics) according to the manufacturer’s recommendations for use on the Leica Bond RXm automated slide stainer and respective Leica Bond reagents (**Supplementary Table 1**). HIER was performed on tissues with ER2 solution (Leica Biosystems) at 97°C for 30 minutes prior to quenching endogenous peroxidase activity with 3% hydrogen peroxide. *Mtb* was identified using RNAscope 2.5 LS Probes B-MTB-23SrRNA-1-C1 (Advanced Cell Diagnostics #471658) and B-MTB-pre-rRNA-O1-C2 (Advanced Cell Diagnostics # 507548-C2) diluted 1:50 in the C1 probe (31, 35). ISH probes were visualized using TSA plus tetramethylrhodamine (Akoya Biosciences) at a dilution of 1:750, TSA plus fluorescein (Akoya Biosciences) at a dilution of 1:1000, and TSA plus cyanine 5 (Akoya Biosciences) at a dilution of 1:750 in 1X TSA Plus Automation Amplification Diluent (Akoya Biosciences). Nuclei were counterstained with DAPI (Advanced Cell Diagnostics). Whole stained lung sections were scanned at 40X magnification using an Olympus VS120 microscope, Hamamatsu ORCA-R2 camera, and Olympus VS-ASW 2.9 software at the Experimental Pathology Facility at CSU. Exposure times were determined using the autoexposure function and adjusted to distinguish specific probe hybridization signal from erythrocyte background.

### 2.8 Multiplex fluorescent image analysis

Whole slide images were imported into Visiopharm image analysis software. Granulomas were identified using a decision forest algorithm trained on intensity and density of all fluorophores. Granulomas identified were confirmed or corrected based on annotations acquired from detection on H&E-stained slides. Each granuloma was then automatically assigned a unique ROI number within each lobe using the enumeration function in Visiopharm. Next, using a threshold-based algorithm, nuclei were detected and annotated based on DAPI signal intensity and given margins to allow for membrane analysis. For IHC, membranes were detected based on a threshold intensity of each fluorophore and assigned to their corresponding marker. For ISH, bacilli were detected based on a threshold intensity of the fluorophore assigned to the 23S rRNA probe, and further designated as replicating or non-replicating based on intensity of the corresponding fluorophore assigned to the pre-rRNA probe. In both IHC and ISH, red blood cell autofluorescence was accounted for based on low DAPI signal intensity combined with inherent combined signals of both FITC and tetramethylrhodamine. Thresholds for all markers were adjusted for any differences in staining or imaging quality and then reviewed for accuracy. Following review, counts for each cell type were quantified using an additional Visiopharm algorithm and exported as .csv files for downstream data analysis.

### 2.9 Statistical analysis

Statistical data analysis was performed in R (version 4.3.1). Data, code, and R packages from this study can be accessed through GitHub (https://github.com/PodellLab/Granuloma_RSRatio_ISH). Data exported from Visiopharm were pre-processed to prepare for downstream analysis in R. Preliminary exploratory statistical and graphical testing were performed to evaluate data distribution and differences between treatment groups. An unpaired t-test was used as a preliminary approach to calculate differences in granuloma burden, CFUs, and ISH RS ratios across treatment groups and infection endpoints.

Next, we fit a regression model to investigate the degree to which the immune cell populations in a granuloma are associated with the number of replicating bacteria in that granuloma, adjusting for the total number of bacteria in the granuloma. To do this, we fit a regression model with the number of replicating bacteria in a granuloma as the outcome variable and with three immune cell populations as potential explanatory variables, while controlling for the timepoint and vaccination status and offsetting by the total number of bacteria. To make model coefficients more interpretable, we scaled immune cell counts by dividing by 500, based on the approximate interquartile range of each of these cell populations across the full study data. Therefore, the estimated coefficients represent an expected change per approximately one interquartile-range change in an immune cell population. Specifically, we fit the following quasi-Poisson regression model, adjusting for potential overdispersion of observed count data from each granuloma and including as an offset the log transformation of total bacterial counts measured by ISH:


$$\log\left(\lambda_{i}\right)=\beta_{0}+\beta_{1}X_{1,i}+\beta_{2}X_{2,i}+\beta_{3}X_{3,i}+\beta_{4}X_{4,i}+\beta_{5}X_{5,i}+\text{log}\;\left(T_{i}\right)$$

Where, for each granuloma i :

-
$\lambda_{i}$ : Expected count of replicating *Mtb*
-
$X_{1,i}$ : Scaled count of B cells
-
$X_{2,i}$ : Scaled count of CD4 cells
-
$X_{3,i}$ : Scaled count of CD8 cells
-
$X_{4,i}$ : Indicator of vaccine status of the mouse with granuloma i , where unvaccinated is 0 and BCG-vaccinated is 1
-
$X_{5,i}$ : Indicator of infection endpoint of the mouse with granuloma i , where day 56 is 0 and day 114 is 1
-
$T_{i}$ : Total count of *Mtb* (replicating and non-replicating) in granuloma i

-
$\beta_{0}-\beta_{5}$ : Model coefficients estimated from the data

## 3 Results

### 3.1 At the whole tissue level, BCG vaccination mediates reduction in culturable *Mtb* burden and total pulmonary pathology burden

**Figure 1:**
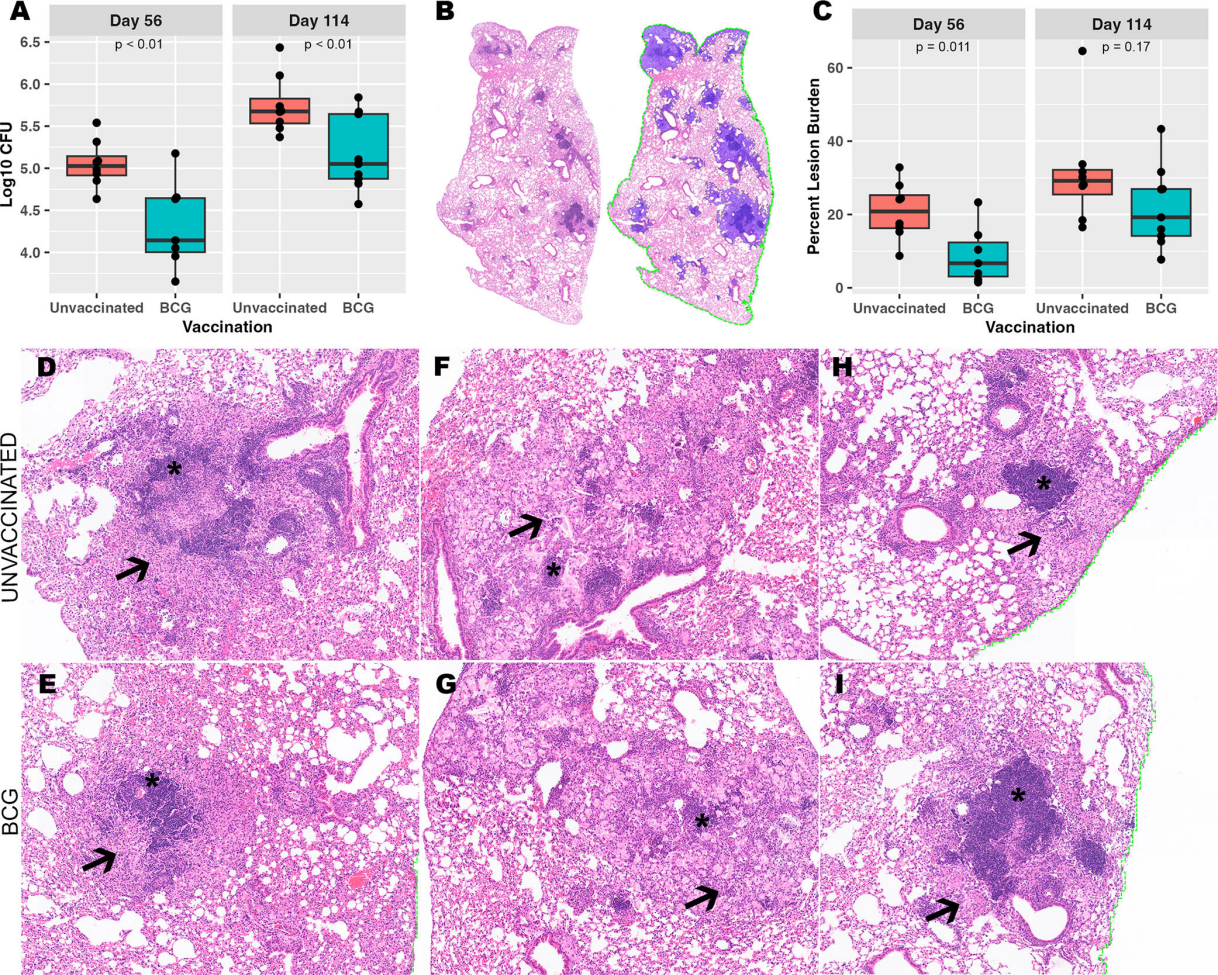
Despite BCG vaccine-mediated reduction in culturable bacterial burden, a heterogeneous granuloma response is shared across both vaccinated and unvaccinated mice. **(A)** Culturable *Mtb* colony forming units (CFU) in BCG-vaccinated and unvaccinated mice from left lung lobe tissue homogenates at day 56 and day 114 post-infection. **(B)** Right caudal lung lobes were H&E-stained after both timepoints of *Mtb* infection. A representative example is provided of a BCG-vaccinated mouse to identify and quantify granuloma lesion burden using Visiopharm image analysis software where granulomas (left) are detected across the entire lung lobe section as shown with blue overlay (right). **(C)** Percent granuloma lesion burden separated by infection endpoint and vaccination status; each point represents a lung section from an individual mouse. **(D-I)** Granulomas with diverse morphology shared between unvaccinated and BCG-vaccinated mice. **(D, E)** Loose centralized aggregates of lymphocytes (asterisk) surrounded by epithelioid macrophages (arrow). **(F, G)** Scattered small lymphocyte aggregates (asterisk) surrounded by heavily vacuolated macrophages, neutrophils and foci of necrosis embedded within areas of alveolar interstitial fibrosis (arrow). **(H, I)** Centralized dense lymphoid aggregates (asterisk) surrounded by vacuolated macrophages (arrow) and discrete granuloma boundaries. Images provided at 50X magnification.

Lung lobes were collected at necropsy at days 56 and 114 post-infection to assess *Mtb* burden at the level of the whole lung tissue. Colony forming units (CFU) of culturable bacteria in lung homogenate increased between day 56 and day 114 post-infection in both unvaccinated and BCG-vaccinated mice. However, BCG-vaccinated mice displayed reduced CFU burden at both days 56 and 114 post-infection compared to unvaccinated mice (**Figure 1A**). To establish whether the BCG vaccination offers protection from progressive pulmonary pathology at days 56 and 114 post-infection, the granuloma lesion burden was quantified histologically across the whole lung lobe using image analysis software (**Figure 1B**). Consistent with CFU at day 56, a significant decrease in granuloma lesion burden was observed in BCG-vaccinated mice compared to unvaccinated mice at day 56. Between day 56 and day 114 post-infection, the overall granuloma lesion burden increased in both groups. A decreased granuloma lesion burden was observed in BCG-vaccinated mice at day 114 post-infection although not statistically significant (**Figure 1C**).

### 3.2 Immune protection is demonstrated in whole lung granuloma lesion burden with histologically divergent features

Regardless of differences across pulmonary pathology burden, heterogeneity could be observed in morphological structure and cell composition among individual granulomas within an individual mouse, within vaccination status, and within infection endpoints (**Figures 1D-I**). H&E-stained lungs demonstrated granulomas with morphology of multiple and variable subtypes as observed by the reviewing pathologist (Podell BK). Granulomas were observed that consist of large centralized lymphoid aggregates resembling *de novo* tertiary lymphoid structures, some of which contain follicular centers composed of cells with macrophage-type morphology. Lymphoid structures are surrounded by either dense and eosinophilic epithelioid macrophages that replace pulmonary architecture (**Figures 1D, E**), or alveolitis consisting of loose aggregates of heavily vacuolated macrophages contained within remnant alveolar spaces (**Figures 1H, I**). Other granulomas consisted of multiple smaller and less organized lymphoid aggregates surrounded by heavily vacuolated macrophages or have a paucity of lymphocyte cells and consist almost entirely of vacuolated macrophages. Among a proportion of the granulomas with poorly organized lymphoid structures, the heavily vacuolated macrophages were surrounded by alveolar interstitial fibrosis embedding small foci of necrosis with a high frequency of neutrophils (**Figures 1F, G**). While measures of granuloma lesion burden offer an indication of immune protection across the whole lung, this fails to encompass the heterogeneity of histological morphology observed across granulomas. This degree of histological heterogeneity suggests that distinct and diverse immune responses exist within and between mice, irrespective of vaccination, and that granuloma targeted analysis could offer greater insight into correlates of protection that cannot be observed through bulk tissue analyses.

### 3.3 Quantification of replicating *Mtb* by ISH RS ratio reveals heterogeneity in immune control of *Mtb* replication across granulomas

**Figure 2:**
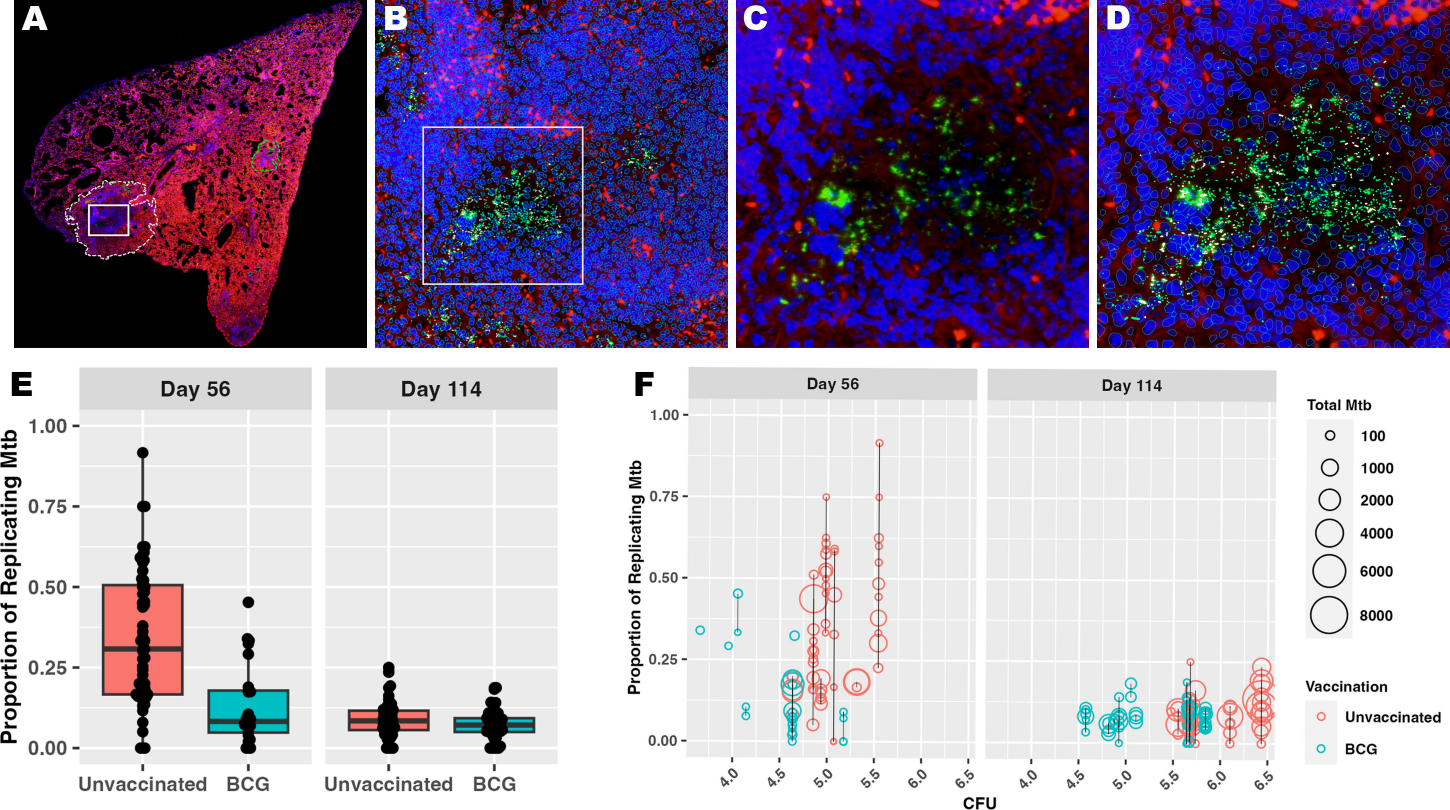
Measuring *Mtb* replication rate by ISH reveals the diversity of granuloma responses contributing to culturable bacterial burden. Populations of *Mtb* bacilli were visualized within each granuloma using RNAScope ISH. All bacteria were identified using probe pairs targeting 23S rRNA, then designated as replicating if the bacillus also contains detectable pre-rRNA with probes targeting the ETS1/ITS1 rRNA sequences. **(A)** Low magnification subgross view of a full lung section from an unvaccinated mouse shows a granuloma designated as a region of interest (ROI) by image analysis (white dotted line); white box designates the granuloma region with detected Mtb rRNA shown in panels **(B–D)**. **(B)** 100X magnification view showing the image analysis detection of all host cells and Mtb bacilli within the region designated in **(A)**. Bacilli can be observed within the inset region designated by the white box. **(C)** 400X view shows 23S rRNA detection representing the bacilli (green), some of which express pre-rRNA (white) that are designated as the replicating population. **(D)** Same field of view as in **(C)** with image analysis labeling of 23S (green) and pre-rRNA (white) among host cell nuclei (blue) **(E)** Bacterial counts (23S rRNA) and proportion of replicating bacilli (23S rRNA + pre-rRNA) are derived using Visiopharm image analysis. Each point reflects the proportion of replicating *Mtb* within a granuloma as measured by ISH and image analysis. **(F)** Proportion of replicating Mtb and total burden of *Mtb* within each granuloma by ISH are shown at the culturable pulmonary bacterial burden for each mouse measured by CFU. Each circle represents a granuloma ROI, and the size of each circle represents total *Mtb* burden by ISH. The black line connecting circles represents a single mouse, where all granulomas detected for that mouse fall along the line and share a common measure of whole lung CFU.

Divergent features of granuloma histomorphology suggest a more granular measure of bacterial replication may distinguish differences between granulomas. To better understand each granuloma’s ability to control bacterial replication, multiplexed fluorescent *in situ* hybridization (ISH), using targets analogous to the RS ratio dPCR (31, 35), was employed to visualize individual replicating and nonreplicating *Mtb* organisms, referred to as RS ratio ISH. In this approach, a replicating bacillus was defined by colocalized detection of both ETS1/ITS1 pre-rRNA sequence and mature 23S rRNA sequence, while a non-replicating *Mtb* bacillus was defined by detection of 23S rRNA only. 191 granulomas identified on H&E-stained slides were evaluated (**Table 1**). The number of granulomas per mouse tended to be higher in unvaccinated mice at day 56 post-infection compared to BCG-vaccinated mice, but increased in number among BCG-vaccinated mice between day 56 and day 114 post-infection. 23S rRNA and ETS1/ITS1 pre-rRNA detection was quantified using Visiopharm image analysis software and the proportion of replicating bacteria relative to total bacteria calculated (**Figures 2A–D**). At both days 56 and day 114 post-infection, granulomas from unvaccinated mice displayed higher proportions of replicating *Mtb* compared to BCG-vaccinated animals (**Figure 2E**). Collectively, the proportions of replicating *Mtb* decreased across granulomas between day 56 and day 114, irrespective of vaccination status. Regardless of timepoint or vaccination status, a wide range of total bacterial burden and replication status was observed between and within groups and within individual animals.

**Table 1:** Granuloma-level measures of host cells and *Mtb*.

	Day 56			Day 114		
	Unvaccinated (*n* = 59)	Vaccinated (*n* = 24)	Overall (*n* = 83)	Unvaccinated (*n* = 55)	Vaccinated (*n* = 53)	Overall (*n* = 108)
Host	Median Count			Median Count		
CD4 T cell	162	90	157	261	449	320
CD8 T cell	169	170	169	219	355	259
B cell	137	95	116	154	387	234
Other	2036	1952	2036	2407	2601	2436
Total	2441	2325	2424	3205	3538	3337
Mtb	Median Count			Median Count		
Replicating	19 (22.6%)	2.5 (5.3%)	13 (18.3%)	28 (7.1%)	13 (6.8%)	15.5 (6.6%)
Nonreplicating	51	37	46	368	183	213.5
Total	84	47	71	393	190	236

Counts of granulomas in each group are indicated by n.

Considering the heterogeneity of replicating proportions of *Mtb* observed across the granuloma, a direct comparison was made to the whole lung CFU per mouse that, as a stand-alone metric, supports vaccine-mediated protection. In comparing these metrics, it is evident that regardless of CFU value, granulomas per mouse vary in number. Within and between mice, these granulomas display an expansive range of total *Mtb* burden and proportion of replicating *Mtb* by ISH. There was no evident directional association between replicating *Mtb* by RS ratio ISH and culturable *Mtb* burden by CFU (**Figure 2F**). Rather, CFUs appear to more linked to the total number of granulomas in the mouse. These data suggest that total CFU burden reflects a collective contribution of diverse granuloma responses with differing capacities to control *Mtb* replication. Overall, the results from granuloma-targeted RS ratio ISH highlights that a wide range of capacity for immune control of *Mtb* replication exists across granulomas, which cannot be effectively captured by CFU. Thus, RS ratio ISH may distinguish granulomas with immune cell composition that provide greater control of *Mtb* replication.

### 3.4 Granulomas exhibit divergent composition of adaptive immune cell phenotypes by IHC

**Figure 3:**
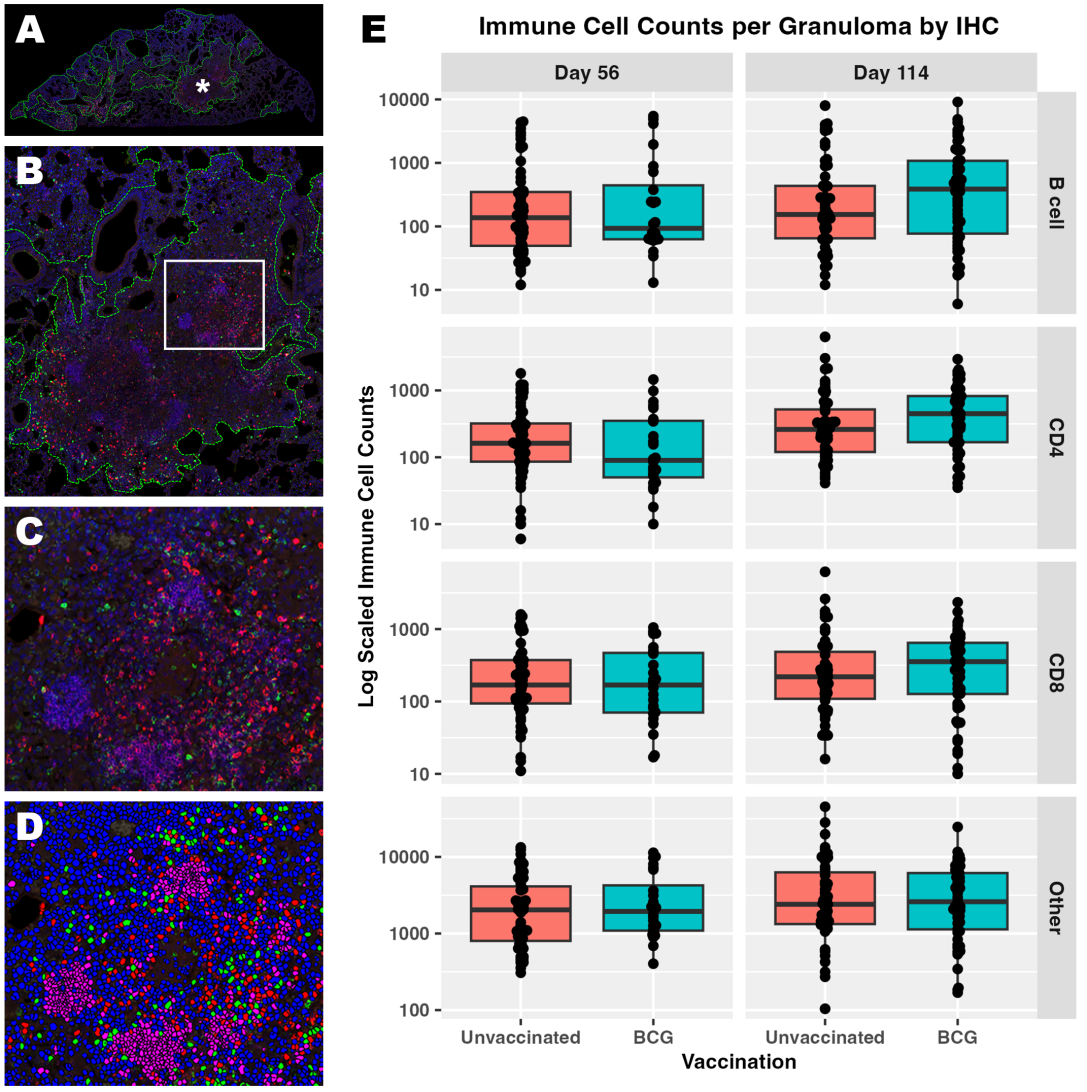
Immune cell composition of granulomas identifies a wide degree of host heterogeneity that alone cannot distinguish trends in immune protection. Populations of CD4, CD8, and B cells were visualized within each granuloma using multiplexed fluorescent IHC with a DAPI nuclear counterstain. **(A)** Low magnification subgross view of a full lung section from an unvaccinated mouse shows granulomas designated as regions of interest (ROI) by image analysis (green dotted lines). **(B)** Higher magnification view of the granuloma ROI designated by the asterisk in **(A)**. **(C)** Immune subsets are demonstrated at 200x magnification within the inset region designated by the white box in B; B cells (pink), CD4 cells (green), CD8 cells (red). **(D)** The same 200X view as in **(C)** is shown with each immune subset or ‘other cell’ designated using image analysis; B cells (pink), CD4 cells (green), CD8 cells (red), other cells (blue, DAPI only). Nuclei detected that do not belong to either CD4, CD8, or B cell are classified as other. **(E)** Cell counts detected at day 56 and day 114 post-infection within each granuloma ROI. Each point represents a granuloma.

We investigated underlying spontaneous and BCG-induced adaptive immunity by multiplexed IHC in conjunction with the granuloma heterogeneity observed both histologically and in *Mtb* replication rate by RS ratio ISH. Essential adaptive immune cell phenotypes were considered because previous work shows a reduction in total lung RS ratio at the onset of adaptive immunity (31). Accordingly, CD4 cells, CD8 cells, B cells, and other host cells were targeted within granulomas in which the RS ratio ISH was quantified (**Table 1**). Within each granuloma designated as an independent ROI, each cell subset was identified and quantified using Visiopharm image analysis software (**Figures 3A-D**). Granulomas displayed heterogeneous cellular composition and organization across all groups and endpoints evaluated, where total cell counts of any measured phenotype are unable to distinguish vaccine status or day of infection (**Figure 3E**). All measured immune cells increase in median number per granuloma between day 56 and day 114, and this increase remained similar across B cells, CD4, and CD8 cells, but these changes between infection endpoints are small in comparison to the large variability observed within each group. The most prevalent cell type at either day 56 or day 114 are those that do not belong to any of the three evaluated phenotypes and are designated by the term, “other cells”. Overall, a wide spread of cell counts was observed independent of timepoint or vaccination status, exemplifying differences across granulomas that may provide context for ability to control bacterial replication.

### 3.5 Relative rate of *Mtb* replication is inversely associated with CD4 and CD8 cell composition at the granuloma level

**Figure 4:**
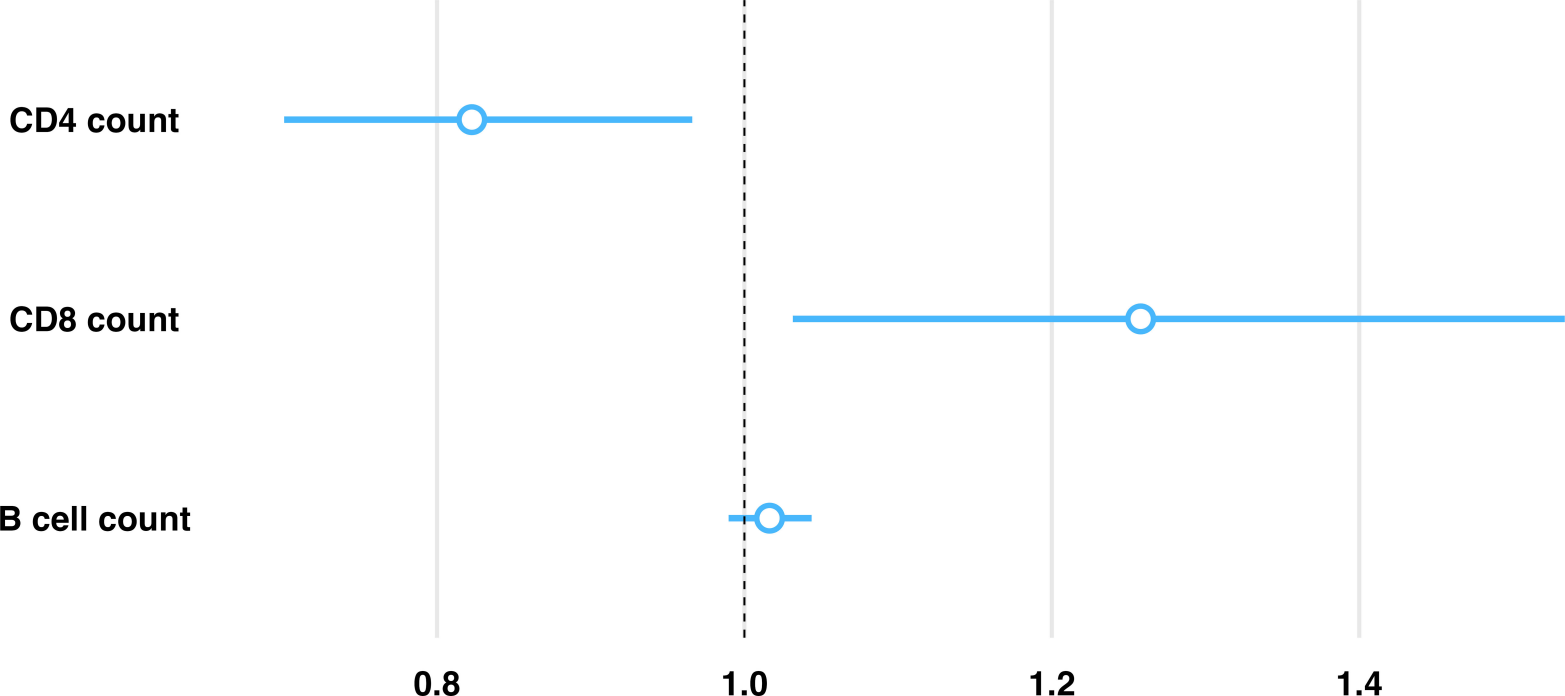
Model predictions between proportion of replicating Mtb and immune cell composition within each granuloma highlights an inverse association with CD4 and CD8 cells. Estimated relative rate of replicating Mtb is shown per 500 cell increase (approximate IQR) of each immune cell subset within an individual granuloma as estimated by a generalized linear regression model, controlling for vaccination status and infection endpoint. Controlling for all other evaluated cell phenotypes, there is an associated 17.7% reduction in the proportion of replicating Mtb for every 500 cell increase in CD4, an associated 25.8% increase in the proportion of replicating Mtb for every 500 cell increase in CD8, and no significant association in the proportion of replicating Mtb for every 500 cell increase in B cells.

Having established granuloma heterogeneity in the C57BL/6 mouse model using simultaneous measures of host and bacterial response, we then performed regression analysis to identify any association with immune environments capable of controlling *Mtb* replication. To do so, a generalized linear regression model was utilized to determine associations between immune cell composition and counts of replicating *Mtb*, while accounting for total *Mtb* burden through a model offset. Based on differences between interquartile ranges (IQR) among immune cells, counts of immune cells were scaled down by a factor of 500 to approximate the IQR, which facilitates interpretation of respective comparisons. Controlling for all variables, irrespective of vaccine status or timepoint, an inverse relationship between CD4 and CD8 cells was observed with changes in the relative rate of bacterial replication. A 17.7% decrease in estimated relative rate of *Mtb* replication was associated with each 500-cell increase in CD4, and a 25.8% increase in relative rate of *Mtb* replication was associated with each 500-cell increase in CD8, while B cell count showed no significant association with *Mtb* replication (**Figure 4**). Controlling for differences in these host cell populations, BCG vaccination was associated with a 35.4% decrease in estimated relative rate of replication compared to unvaccinated, while progression to day 114 post-infection was associated with a 54.7% decrease over time compared to day 56 post-infection. Here we identify a significant association between granuloma immune cell composition and control of *Mtb* replication. Data obtained from all evaluated granulomas, including immune cell counts and replication rates are available at https://github.com/PodellLab/Granuloma_RSratio_ISH.

### 3.6 Histologic morphology of granulomas is consistent with immune cell counts and rate of Mtb replication

**Figure 5:**
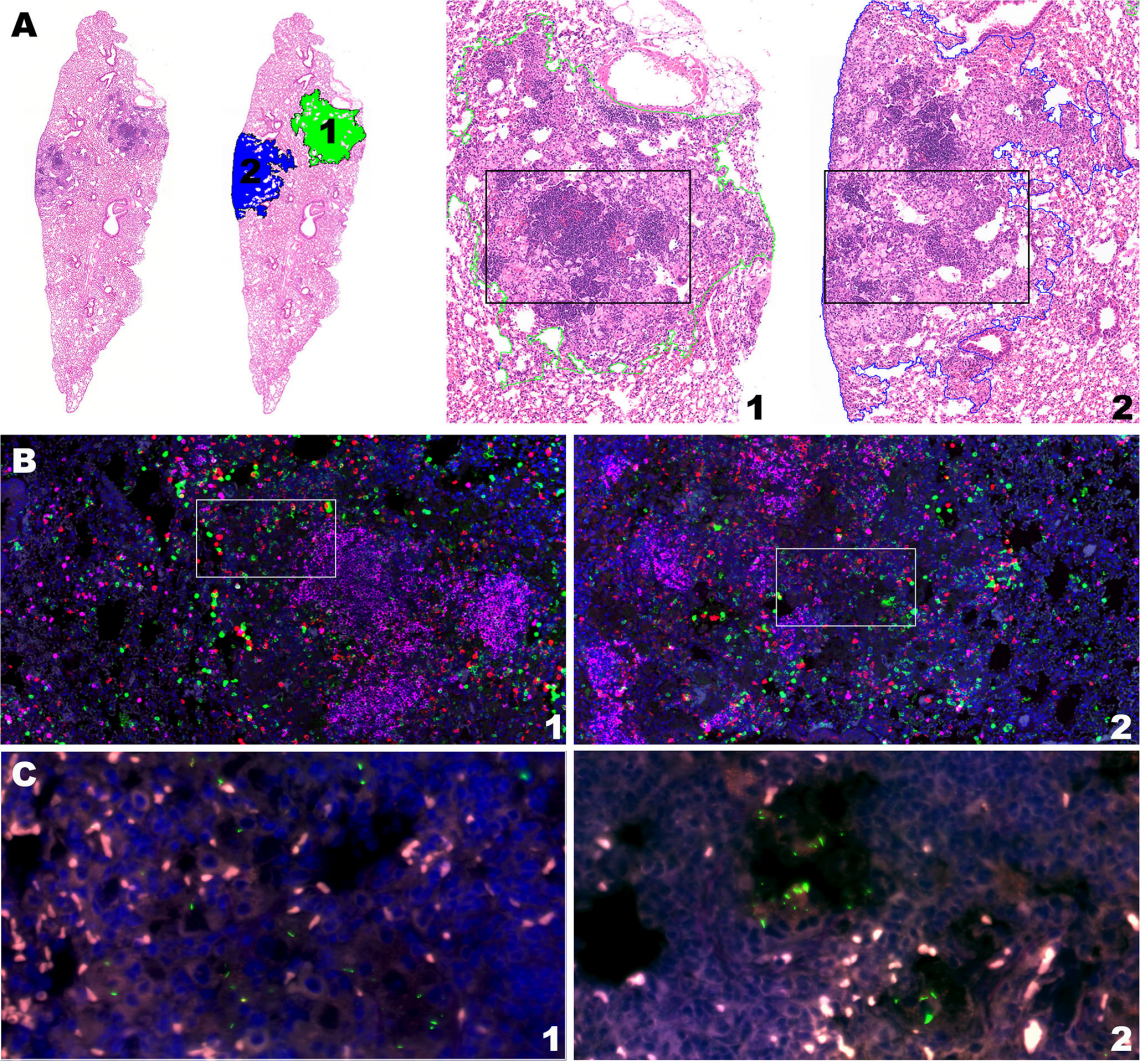
Intramouse heterogeneity in granuloma morphology, immune cell composition, and Mtb replication rate aligns with model predictions. Two neighboring granulomas within the same lung section of the same BCG-vaccinated mouse are shown by H&E stain, multiplexed IHC, and RS ratio ISH. **(A)** H&E stain at subgross showing the ROI of granuloma 1 and 2 from the BCG-vaccinated mouse (10X magnification) at day 56 post-infection and H&E morphology of each granuloma outlined by green and blue dotted lines for granulomas 1 and 2, respectively (50X magnification). **(B)** Multiplexed IHC demonstrating populations of CD4 cells (green), CD8 cells (red) and B cells (pink) in granulomas 1 and 2 (100X magnification). White boxes indicate inset regions for visualization of Mtb organisms. **(C)** Mtb bacilli detected with pre-rRNA and 23S rRNA probes by RNAScope ISH in granulomas 1 and 2.

Based on predictions using this model, we back-translated associations between cell composition and RS ratio ISH replication rates to evaluate granuloma morphology associated with immune control of bacterial replication. Selection of two granulomas from the lung lobe of a single BCG-vaccinated mouse at day 56 post-infection highlights the observation that different degrees of bacterial replication existing between granulomas is associated with immune cell composition unique to each granuloma (**Figure 5**). Between these two granulomas within this single mouse, CD8 cells differ by 547 cells and Mtb replication is increased by 28.9% (**Table 2**). Consistent with the multiple morphologies described in **Figure 1**, these granulomas contain either higher proportions of vacuolated macrophages or epithelioid macrophages and B cell-rich *de novo* follicular structures. In these granulomas, the higher Mtb replication rate is localized to macrophages with vacuolated morphology, which is overrepresented in the granuloma with the higher CD8 count. These findings indicate that cell populations with a designated morphology may align with poor control of Mtb replication. Furthermore, granuloma morphology may indicate an immune environment with capacity to successfully limit, or fail to control, Mtb replication.

**Table 2:** Individual granuloma measures informing histopathology morphological features.

	BCG 24–1	BCG 24–2
Proportion replicating Mtb	0.142	0.183
Replicating Mtb	27	43
Nonreplicating Mtb	163	192
Total Mtb	190	235
CD8 count (%)	774	1321
CD4 count (%)	1082	1027
B220 count (%)	3286	3485
Other cell count (%)	9097	11638
CFU (Log_10_ per g tissue)	5.05	5.05

Examples of granulomas highlighting histopathological morphology based on GLM associations are shown in **Figure 5**. Characteristics of each of granuloma are identified by individual mouse-granuloma (i.e., 24–1). Data for all granulomas evaluated in this study are available on the GitHub repository.

## 4 Discussion

In this study, we applied the RS ratio through *in situ* hybridization (RS ratio ISH) to identify the frequency of new rRNA production, which serves as a proxy measure of *Mtb* replication (31, 35). Here, we identified the replicative response of *Mtb* to immune pressure over two chronic endpoints of infection and in response to BCG vaccination. A wide range of *Mtb* replication states was demonstrated across granulomas both within individual animals, as well as between animals evaluated at the same infection endpoint, independent of respective vaccination status. These results highlight the extensive degree of heterogeneity among *Mtb* replicative states across granulomas. We found that culturable bacterial burden from whole lung homogenate, as determined by CFU, does not necessarily reflect the spectrum of *Mtb* replication states across granulomas. Although vaccination does reduce CFU, the difference in *Mtb* replication rate remains similar in some granulomas of BCG-vaccinated mice, compared to unvaccinated mice. Additionally, these data indicate that rate of replication within a granuloma and total bacterial burden do not necessarily correlate. In many instances, granulomas with the highest rate of *Mtb* replication do not have the highest burden of bacteria, as identified by detection of 23S rRNA alone. These data suggest that a differential host response may exist between granulomas with high or low replication rates, or those with high or low *Mtb* burden. We hypothesized discrimination of these immune environments and their overarching contribution to the replicative state of the *Mtb* bacilli may identify correlates of protective responses.

We identified a wide diversity of granuloma compositions, containing varying frequencies of CD4, CD8 and B cell populations, which is consistent with the highly heterogeneous replication rates of *Mtb* across granulomas. These findings are inconsistent with the constrained host immune response characteristic of the C57BL/6 mouse strain. These mice have a well-documented Th1 skewed cell-mediated immune response with granulomas described as lacking advanced pathology features (3). Despite an apparent uniformity in the immune response to *Mtb* infection, our findings indicate that granuloma heterogeneity exists in C57BL/6 mice, highlighting the importance of granuloma-targeted analysis. This approach provides an adjunctive method to distinguish granuloma-specific differences in immune response within and between animals enabling determination of the local granuloma response as opposed to whole tissue measures, such as flow cytometry.

To determine if the variability in *Mtb* replicative state is associated with differences in host response, we established a generalized linear regression model to evaluate the association between *Mtb* replication rate and CD4, CD8 and B cell populations across nearly 200 granulomas. Based on culturable CFU, these broad phenotypic categories have an established role in TB disease as a primary outcome measure. However, due to previous limitations in concurrent host and pathogen *in situ* analyses, determining associations between each immune cell subset and the *Mtb* bacterial replication state was not achievable. Using multiplexed IHC and RS ratio ISH to assess both host phenotype frequency and bacterial replication rate, we determined the association between immune cell composition and the respective response of the bacterial population within each granuloma. Through this, a unique association between CD4 and CD8 cell frequencies and *Mtb* replication rates has been established in any individual granuloma, while a statistically significant association between B cells and *Mtb* replication rate was not observed in the evaluated granulomas.

Our model indicated that every unit increase in CD4 cells was associated with a significant decrease in *Mtb* replication rate, while every unit increase in CD8 cells was associated with a significant increase in *Mtb* replication rate. Although CD4 and CD8 cells have established roles in the constraint of *Mtb* growth in tissue, the opposing directionality of each cell type association may suggest different roles for CD4 and CD8 cells in the granuloma response. However, this relationship does not yet indicate any directional causality despite definitive associations. In this context, the RS ratio measure of replication rate may inform further investigation of granuloma response where further interventions that manipulate specific host cell phenotypes are needed to determine the mechanism by which a designated cell type contributes to immune control of *Mtb* replication.

In concordance with the granuloma-level degree of heterogeneity in both host and bacterial populations, differences in granuloma morphology based on traditional H&E-stained slides were observed. In multiple conventional inbred mouse strains, including the C57BL/6 mouse, tertiary lymphoid follicular structures are a frequent and dominant feature of the response to *Mtb* infection (36, 37). Granulomas can be distinguished as having either dense and well-structured follicles, or loosely organized lymphoid infiltration. Further, the innate populations of macrophages may be either heavily vacuolated or more epithelioid in appearance. Vacuolated macrophage populations may contain varying levels of accompanying neutrophils and cellular fragmentation consistent with multiple mechanisms of macrophage cell death. We found that intragranuloma follicular structures are heavily dominated by B cells and have limited T cell involvement. Although some granulomas have high proportions of follicle-associated B cells, our model indicated no statistical association with *Mtb* replication rate. In contrast, we histologically observed that granulomas with high CD4 T cell frequencies and lower *Mtb* replication rates contained more epithelioid macrophage populations. Granulomas with high CD8 T cells and high *Mtb* replication rates contained prominent vacuolated macrophage populations. Further *in situ* investigation of unique H&E-defined granuloma morphology may reflect critical microenvironmental differences in host response, including host immune cell composition, and subsequently constraint of *Mtb* replication (38).

The key associations in immune cell composition and bacterial replication rate revealed by our model leads us to ask the question ‘does restriction of *Mtb* replication represent an ideal pathogen-targeted measure of immune protection?’. *Mtb* has an inherent capacity to enter a dormancy-like phenotype, and previous studies using progressive hypoxia models of *in vitro* grown *Mtb* suggests that dormancy onset accompanies a reduction in replication (31, 39). Such dormant phenotypes may contribute less to progressive TB disease (40, 41). Increased dormancy induced by immune pressure on the *Mtb* bacilli may be associated with an altered treatment response (42) or may reflect a temporary replicative constraint on the *Mtb* population (43), which may recover following changes in the immune environment. Alternatively, a reduced replication rate could reflect improved control of bacterial growth and an increase in bacteriostatic effect from the ongoing immune response (44–46). Distinguishing dormancy from other mechanisms of immune constraint on Mtb replication requires further investigation of the bacterial response *in situ*. This presents its own challenges but may be determined by emerging techniques, such as SearchTB, using *Mtb* enriched extracts from tissue (47).

There are limitations to this study and this method. The evaluation of only three adaptive immune cell phenotypes, B cells, CD4 and CD8 T cells, underrepresents the complexity of the TB granuloma (48). This is evident based on recent single cell RNA seq studies that have demonstrated 18 distinct lymphoid cell sub-populations involved in the pulmonary granulomatous response of murine TB models (20). We recognize the power of adjunctive immunological methods (i.e. flow cytometry, scRNAseq) in assessing host protection as well as the greater capacity of these methods to identify specific details of the host response. However, this study serves as proof-of-concept that granulomas with differing cell compositions also have distinct differences in capacity to influence *Mtb* replication. Additionally, the results presented here represent only one context of a modeled *Mtb* infection, that of unvaccinated and BCG-vaccinated C57BL/6 mice infected by low-dose aerosol exposure to the Erdman strain. The application of this approach to other model strains, species, vaccine compositions, and strains of *Mtb* will further elucidate the capacity for granuloma targeted analysis to identify correlates of immune protection. The RS ratio used here provides adjunctive value to whole tissue measures, with the distinct advantage of evaluating the concurrent host and *Mtb* bacterial response *in situ* within any given granuloma. The RS ratio ISH method is not intended to serve as an alternative or replacement for CFU determination as a measurement of TB disease outcome. Rather, this approach offers orthologous information to culturable bacterial burden by providing critical information on the *Mtb* replicative state in the native granuloma microenvironment that cannot be measured by CFU alone (31). The ongoing development and implementation of complementary granuloma and whole lung methodologies holds promise for advancing our understanding of TB and facilitating development and assessment of effective vaccines and therapeutics.

## Data Availability

The datasets presented in this study can be found in online repositories. The names of the repository/repositories and accession number(s) can be found below: https://github.com/PodellLab/Granuloma_RSratio_ISH.
