# Supplementary material for: Heterogeneity in immune cell composition is associated with Mycobacterium tuberculosis replication at the granuloma level
Source: Front Immunol. 2024 Aug 26;15:1427472. doi: 10.3389/fimmu.2024.1427472 (PMC11381408; doi:10.3389/fimmu.2024.1427472)
Supplement: Supplementary file 1 [file Table1.docx]

Supplementary Material

**Supplemental Materials and Methods.**

**Supplemental Table 1. Reagent information**

| **Reagent** | **Cat. Number** | **Supplier** |
| --- | --- | --- |
| 2.5% Normal Goat Serum Blocking Solution | S-1012-50 | Vector Laboratories |
| CD4 (Clone 4SM95) | 14-9766-82 | eBioscience |
| CD8a (Clone 4SM15) | 14-0808-82 | eBioscience |
| CD45R/B220 (Clone RA3-6B2) | 103201 | BD Biosciences |
| Goat Anti-Rat IgG | MP-7404 | Vector Laboratories |
| Opal 520 | Akoya, FP1487001KT | Akoya |
| Opal 570 | Akoya, FP1488001KT | Akoya |
| 1X Plus Automation Amplification Diluent | FP1609 | Akoya |
| TSA Plus Cyanine 5 (Cy5) | NEL745001KT | Akoya |
| TSA Plus Fluorescein (FITC) | NEL741001KT | Akoya |
| TSA Plus Tetramethylrhodamine (TMR) | NEL742001KT | Akoya |
| Spectral DAPI | FP1490 | Akoya |
| RNAscope 2.5 LS Probe B-MTB-23SrRNA-1-C1 | 471658 | ACD Bio |
| RNAscope 2.5 LS Probe B-MTB-pre-rRNA-O1-C2 | 507548-C2 | ACD Bio |
| RNAscope LS Multiplex Fluorescent Reagent Kit | 322800 | ACD Bio |
| RNAscope LS 4-Plex Ancillary Kit Multiplex Reagent Kit | 322830 | ACD Bio |
| VECTASHIELD Vibrance® Antifade Mounting Medium | H-1700-10 | Vector Laboratories |
| ER1 | AR9961 | Leica Biosystems |
| ER2 | AR9640 | Leica Biosystems |
| Wash Solution 10X | AR9590 | Leica Biosystems |
| Dewax Solution | AR9222 | Leica Biosystems |
| Leica Research Kit Wand | DS9777 | Leica Biosystems |
